# Osteonecrosis of the hip: is there a difference in the survivorship of total hip arthroplasty with or without previous vascular iliac bone grafting?

**DOI:** 10.1186/s13018-021-02332-6

**Published:** 2021-04-08

**Authors:** Wai-Wang Chau, Jonathan Patrick Ng, Hiu-Woo Lau, Michael Tim-Yun Ong, Kwong-Yin Chung, Kevin Ki-Wai Ho

**Affiliations:** 1 Department of Orthopaedics and Traumatology, Chinese University of Hong Kong, Hong Kong, SAR China; 2 Department of Orthopaedics and Traumatology, Prince of Wales Hospital, Shatin, Hong Kong, SAR China

**Keywords:** Osteonecrosis of the femoral head, Survivorship, Hip, Arthroplasty, Vascular iliac bone graft

## Abstract

**Background:**

Osteonecrosis of the femoral head (ONFH) is a debilitating condition. Vascularized iliac bone graft (VIBG) is a joint-preserving surgery to improve blood supply to the avascular portion of the femoral head which may delay secondary osteoarthritis and total hip arthroplasty (THA). However, whether VIBG will affect the subsequent THA survivorship and outcomes are still uncertain.

**Methods:**

Implant survivorship and clinical outcomes were compared between 27 patients who had undergone prior VIBG and 242 patients who had only undergone THA for ONFH. Baseline characteristics and the postoperative Harris Hip Score (HHS) were also recorded and compared between the two groups. Implant survivorship was determined using Kaplan-Meier survival analysis.

**Results:**

The overall implant survival for all patients who had a primary diagnosis of ONFH and eventually underwent THA was 92.9%. There was no significant difference in the implant survivorship between the group who directly received THA (survivorship of 93%) and the group which failed VIBG and was subsequently converted to THA (survivorship of 91.9%) (*p* = 0.71). In addition, higher THA revision rates were associated with smokers and drinkers.

**Conclusions:**

VIBG may be a reasonable option as a “buy-time” procedure for ONFH. Even if conversion to THA is eventually required, patients may be reassured that the overall survivorship and clinical outcomes may not be compromised. Patients are recommended to give up smoking and binge drinking prior to THA to increase implant survival rate.

## Introduction

Symptomatic osteonecrosis of the femoral head (ONFH) is a debilitating condition that has a poorly understood pathogenesis [1]. The aetiology of ONFH is believed to be a combination of genetic predisposition, metabolic factors, and local factors affecting blood supply to the femoral head [2, 3]. For early stages of this disease, a joint-preserving approach is adopted, including pharmacological agents, core decompression with or without adjuctive biological agents, extracorporeal shock wave therapy, bone marrow-derived cell therapies combined with core decompression, non-vascularised or vascularised bone grafting (VIBG), and resurfacing arthroplasty [4–16]. However, once collapse occurs, the only definitive treatment is total hip arthroplasty (THA) [17–19].

In contrast to the smaller proportion of ONFH in Sweden (< 5%) [20] and in the USA (7%) [21] in patients undergoing hip replacement, a local study showed that 45.6% of all THA were performed for patients who had a primary diagnosis of ONFH [22]. Furthermore, ONFH most commonly affects younger patients, with an average age of 33 to 38 years at treatment and is the commonest indication for total hip arthroplasty in this population [23, 24]. In view of this, primary THA as a treatment is often not expected to outlive the patient’s lifespan. Therefore, procedures such as VIBG have been developed in an attempt to save the femoral head, or at least slow down the rate of progression of ONFH before its collapse. In a European study, 42% of patients reported good and excellent results after vascularized iliac crest graft [25]. In a Japanese study of 14 patients who underwent VIBG between 1992 and 2002, 12 of 17 hips (71%) had no disease progression to a more advanced stage in by a mean of 51 months [26]. Therefore, VIBG is a reasonable “buy-time” procedure to delay or even obviate the need for THA. On the other hand, VIBG converting to THA has been reported with different reasons. These include asymmetric bone healing and non-union between the graft and the necrotic subchondral bone in the weight-bearing area, and failure of revascularization of vascularized fibular graft [27, 28]. However, there is a paucity of literature reporting the outcomes of THA in patients who have undergone previous joint-preserving procedures.

The aims of the study was to (1) assess the overall long-term survivorship of THA for ONFH, (2) compare the long-term implant survivorship of THA in patients who had previous VIBG versus THA without previous joint preservation surgeries and (3) compare the long-term clinical outcomes of THA in patients who had previous VIBG versus THA without previous joint preservation surgeries.

## Materials and methods

Joint registry data from a tertiary referral joint replacement centre were retrieved, and a retrospective cohort study of all ONFH patients who underwent THA between 1987 and 2019 was conducted. Ethics approval was obtained from the institutional ethics review committee (ethics approval number 2020.183).

A total of 269 patients (339 hips) were recruited. Patients were stratified into two groups based on whether VIBG had been performed prior to THA (i.e. VIBG+THA vs THA only). Patients who underwent THA for avascular necrosis of the femoral head with at least five years of follow-up were included. The exclusion criteria were (1) patients who underwent core decompression surgery only, (2) patients with previous avascular bone graft surgical treatment, (3) patients clinically diagnosed with inflammatory arthritis and (4) patients with vasculitis conditions. Severity of ONFH was graded according to Ficat and Arlet staging [29]. The procedure and surgical technique for VIBG have been reported in previous literature [30]. All primary THA were performed either through a posterior or anterolateral approach. For those with prior VIBG who later received THA, THA were also performed either through a posterior or anterolateral approach, with cementless femoral and acetabular components.

Baseline characteristics in terms of age, sex, side and risk factors were extracted from electronic patient records. Patients who underwent THA visited the outpatient clinic at the postoperative 1, 3, 6, 12, 18 and 24 months and annually thereafter. Patients were examined and assessed by evaluating the Harris Hip Score (HHS) [31] at each visit. Additionally, medical and surgical complications during earlier visits were also recorded.

The end point of survivorship was defined as revision THA for any cause. Revision THA included any hip exploration following THA (with or without previous VIBG) including exchange of the acetabular or femoral component for aseptic or septic reasons, including component malalignment, osteolysis and component failure.

### Statistical analysis

Baseline characteristics were compared using the Student’s *t* test or Chi-square test where appropriate. Kaplan-Meier (K-M) product-limit method and mean and standard error of survival time estimates were presented. Censorship of K-M curve was defined as the THA survivorship which turned out to be the implant survivorship (i.e. endpoint as THA revision) for both groups. Post hoc Bonferroni comparisons were carried out and presented using the log-rank test. Data analysis were carried out using IBM SPSS 26.0 (Armonk, New York). A two-sided *p* value ≤ 0.05 was considered statistically significant.

## Results

A total of 269 patients (339 hips) were recruited. Of which, 27 patients (37 hips) with previous VIBG underwent conversion to THA (i.e. VIBG+THA group), and 242 patients (302 hips) underwent THA directly with no prior salvage procedure (i.e. THA only). Five patients from the VIBG+THA group underwent bilateral VIBG, then subsequently converted to bilateral THA. In the VIBG+THA group, the mean age of patients receiving VIBG was 38.7 years old, and the mean age of VIBG failure with subsequent THA was 47.5 years old (Table 1). In the THA-only group, patients received THA at a mean age of 58.1 years. The mean age of the patients who underwent THA was significantly higher in the THA-only group (*p* < 0.01). Male gender and steroid treatment were independent risk factors for failure of VIBG (male: odds ratio and 95% CI = 3.63 (1.27, 10.31), *p* = 0.01; steriod treatment: odds ratio and 95% CI = 4.38 (1.14, 16.80), *p* = 0.03) and subsequent conversion to THA (male: odds ratio and 95% CI = 2.70 (1.20, 6.11), *p* = 0.01; steriod treatment: odds ratio and 95% CI = 1.20 (1.01, 1.44), *p* = 0.01).

**Table 1:** Baseline characteristics of patients came across both VIBG and THA (“VIBG+THA” group) (*N* = 27) and those with a primary THA (“THA only” group) (*N* = 242)

Baseline characteristics	VIBG+THA (*N* = 27)	THA only (*N* = 242)	*p* value
Age at VIBG	38.73 ± 9.96 (19, 56)	-	-
Age at THA	47.47 ± 12.06 (29, 78)	58.06 ± 13.12 (21, 84)	< 0.01
Sex			
Male	21 (77.8)	138 (57.0)	0.01
Female	6 (22.2)	104 (43.0)	
Side			
Left	13 (48.1)	120 (49.6)	1.00
Right	14 (51.9)	122 (50.4)	
Risk factors			
Drinking/Smoking	14 (51.9)	123 (50.8)	< 0.01
Steroid	7 (25.9)	23 (9.5)	
Trauma	4 (14.8)	29 (12.0)	
Others	2 (7.4)	67 (27.7)	

*VIBG* vascularized iliac bone graft, *THA* total hip arthroplasty

The mean follow-up years in VIBG+THA patients from VIBG to last seen were 22.1 years. The mean number of years from VIBG to conversion to THA was 9.3 years. The mean follow-up was 11.8 years for the VIBG+THA group, and 11.7 years for the THA group (*p* = 0.95). The mean Harris Hip Score in the VIBG+THA group was significantly higher than the THA-only group (92.63 vs. 81.83, *p* = 0.05). The percentages of THA revision were 8.1% in VIBG+THA patients and 7.3% in THA only patient, and the comparison was not statistically different (*p* = 0.74).

**Fig. 1:**
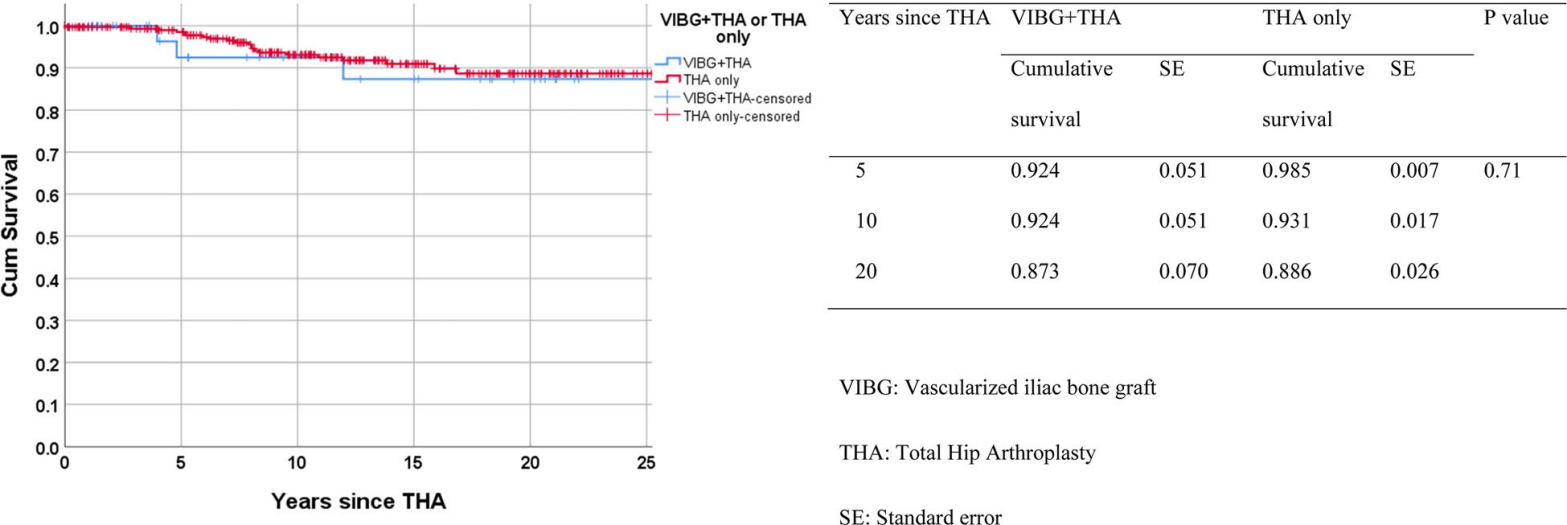
Kaplan-Meier overall survival on patients by group. VIBG, vascularized iliac bone graft; THA, total hip arthroplasty; SE, standard error

**Fig. 2:**
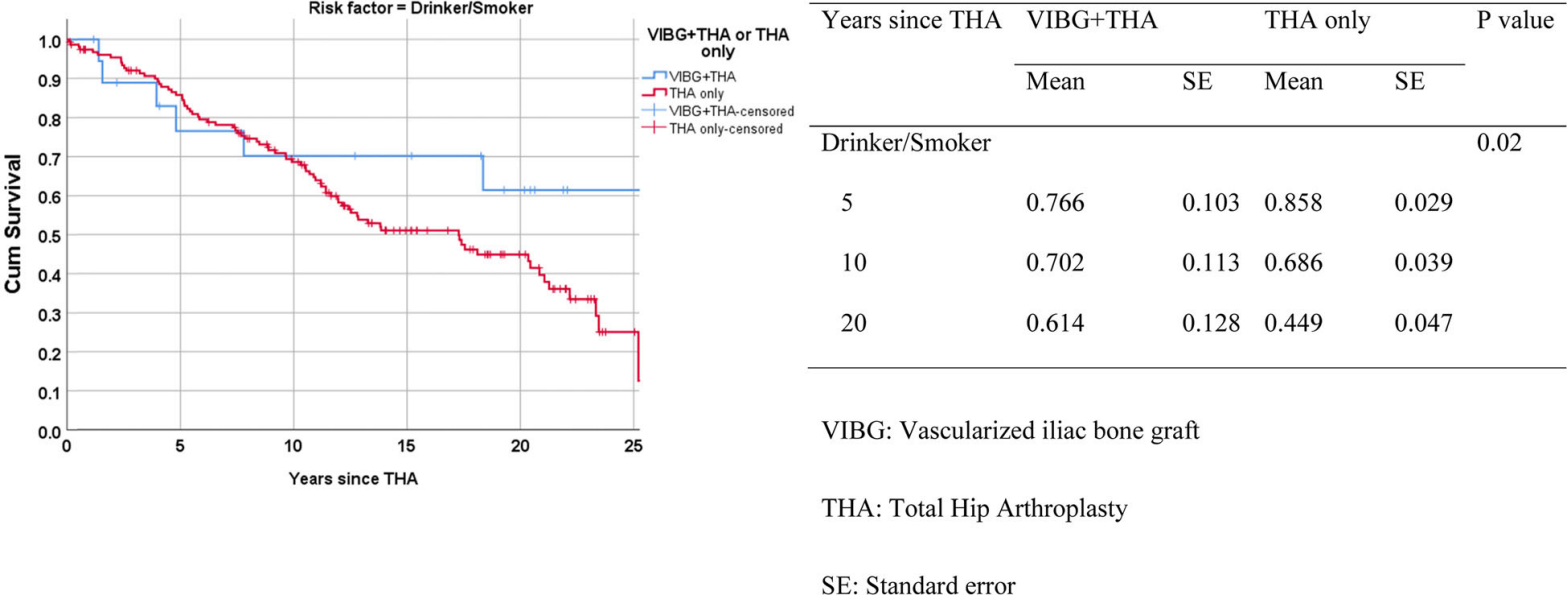
Kaplan-Meier overall survival on patients by risk factors (drinker/smoker), VIBG, vascularized iliac bone graft; THA, total hip arthroplasty; SE, standard error

The overall implant survival for all patients who had a primary diagnosis of ONFH and eventually underwent THA was 92.9% (*N* = 315 of 339). Overall, there were 24 patients who underwent revision THA for aseptic loosening. Twenty-one revisions were in the THA-only group (survivorship of 93%). In contrast, the overall survivorship in the VIBG+THA group was 91.9%, with 3 patients undergoing revision surgery (two separate revisions for acetabular loosening and one revision for femoral loosening). The 5th, 10th and 20th survival rates were similar in both VIBG+THA and THA-only groups (5th year, 92.4% vs. 98.5%; 10th year, 92.4% vs. 93.1%; 20th year, 87.3% vs. 88.6%), and there were no significant differences in the overall implant survivorship between these two groups (*p* = 0.71) (Fig. 1). Considering the effects of risk factors on the implant survival rates, patients who drank or smoked were more likely to have their implant revised after 10 years of THA (implant survival rates at the 10th year = 70.2% (VIBG+THA) vs. 68.6% (THA only), and at the 20th year = 61.4% (VIBG+THA) vs. 44.9% (THA only), overall *p* value = 0.02) (Fig. 2). Patients who smoked or drank had significantly worse overall implant survivorship (mean years 23.14 for smokers or drinkers versus 30.48 in non-smokers or drinkers, *p* < 0.01). Reasons of much lower overall survivals of drinkers or smokers in the THA-only group at 20 years and later compared with the VIBG+THA group were (1) wound discharge (25.9%), (2) loosenings (cup loosening = 9.9%, stem loosening = 6.2%, aseptic loosening = 6.2%), (3) urinary tract infection (7.4%), (4) eccentric wear acetabular cup = 4.9% and (5) others, for example, deep vein thrombosis, gouty attack and intraoperative crack fractures.

## Discussion

There is a paucity of literature reporting the outcomes of THA in patients who have failed prior joint preservation surgeries. In particular, this was the first study to assess whether previous VIBG or associated risk factors would affect the results and survivorship of subsequent THA for patients with ONFH. The most important finding from this study was that the THA implant survivorship was not affected by previous VIBG procedure. In fact, the mean HHS appeared to be better in those have had previous VIBG. However, this must be interpreted with caution as those in the VIBG+THA group are generally younger.

Results from our study are in agreement with previous studies reviewing the outcomes of THA in patients who have undergone previous hip preservation surgeries. Issa et al. compared the implant survivorship, HHS and radiographic outcomes between 92 hips who had undergone prior hip-preserving procedures and 121 hips who had only undergone THA [32]. They found similar survivorship at a mean follow-up of 75 months (93% in prior hip preservation surgery group and 98% in THA-only group). In addition, there were no significant differences in the mean HHS of patients who had previous femoral neck-preserving procedures compared to those who had only undergone total hip arthroplasty. However, none of the patients had undergone VIBG in their cohort. Previous literatures have also reported outcomes of THA with other previous hip preservation procedures, such as rotational osteotomies or resurfacing arthroplasties. Kawasaki et al. evaluated the clinical and radiographic outcomes of 15 hips that initially underwent transtrochanteric rotational osteotomy and later converted to THA. When compared with a matched control group of 16 hips, they found no significant difference in the HHS and survival rates [33]. McGrath et al. compared clinical outcomes of 39 hips who had surface replacement arthroplasties converted to standard total hip arthroplasty with a matched group of 39 hips who had standard THAs [34]. At a mean follow-up of 45 months, they reported similar implant survivorship and mean HHS between two groups. In contrast, Ferle et al. proposed that previous strut grafting prevented optimal positioning of the femoral stem and canal fit, especially for a cementless THA [35]. In their series of 13 cementless THA at a minimal follow-up of 2 years, they reported suboptimal alignment of the femoral component, with two hips requiring revision for femoral stem loosening. Berend et al. also suggested that vascularised fibular grafting altered the biomechanics of the hips, which led to more difficult subsequent THA conversion. In their series of 89 hips who underwent THA for failed vascularised fibular grafting, they reported an overall implant survivorship of 82% at a mean follow-up of 9 years [36]. In addition, 50% of revised hips had required multiple revisions.

We also investigated the effects of different risk factors on THA survivorship and found that percentages of implant survival of ONFH patient who underwent primary THA were much affected (i.e. much smaller) than patients that underwent VIBG+THA if they smoked or drank. Smokers or drinkers had worse implant survival in both the THA only and VIBG+THA groups. A systematic review carried out in Minneapolis found that smoking was associated with significantly higher risk of postoperative complication following THA, and the effect was regardless whether the patients were current smokers (risk ratio, 1.24 (1.01 to 1.54)) or ex-smokers (risk ratio, 1.32 (1.05 to 1.66)) [37]. Complications arisen from smoking on implant outcome after THA had also been reported in a population register-based case-control study (1997) [38], another meta-analysis of cohort studies conducted later (2015) [39] and a larger scale population-based cohort study (2019) [40]. Risk factors considered in this K-M survival comparisons were smoking or drinking, which means drinking is another risk factor, complementary or independently, affecting implant survival. As a result, smoking or drinking had a much bigger impact on the implant survival in the THA-only group than the VIBG+THA group. This finding has not been reported before. Smoking cessation and stopping binge drinking should be encouraged for ONFH patients who suggested to undergo THA and after THA in order to increase implant survival rate.

### Limitations of this study

There are several limitations to our study. Firstly, it is a retrospective cohort study with relatively small sample size, albeit the largest cohort of long-term follow-up of THA with previous VIBG in the literature. In addition, patient reported outcomes and broader quality of life measures were not evaluated. However, the authors believe that the data presented are valuable to clinicians in choosing and discussing the management options for patients with ONFH. Moreover, we did not include other hip preservation surgeries in our study as our centre was the major regional institution performing VIBG in ONFH patients, inclusion of a relatively smaller number of patients who underwent other hip preservation surgeries would have resulted in more heterogenous data. Future studies that use pooled data from multiple institutions may be useful to better evaluate and draw conclusions on the survivorship and outcome of THA with prior hip preservation surgeries as a whole.

## Conclusion

This study reported an excellent overall survivorship at a mean follow-up of 11.8 years, regardless of whether the patient had undergone previous VIBG. In light of these findings, the authors believe that VIBG may be a reasonable option as a “buy-time” procedure for ONFH. Even if conversion to THA is eventually required, patients may be reassured that the overall survivorship and clinical outcomes may not be compromised. Apart from drinking, smoking is also another risk factor associated with worsening implant survival rate, particularly in patients underwent THA only.

## Data Availability

The datasets used and/or analysed during the current study are available from the corresponding author on reasonable request.

## Abbreviations

ONFH: Osteonecrosis of the femoral head
VIBG: Vascularized iliac bone graft
THA: Total hip arthroplasty
HHS: Harris Hip Score
K-M: Kaplan-Meier
CI: Confidence interval
